# Functional significance of DNA methylation: epigenetic insights into Sjögren’s syndrome

**DOI:** 10.3389/fimmu.2024.1289492

**Published:** 2024-03-06

**Authors:** Yanqing Wang, Farooq Riaz, Wei Wang, Jincheng Pu, Yuanyuan Liang, Zhenzhen Wu, Shengnan Pan, Jiamin Song, Lufei Yang, Youwei Zhang, Huihong Wu, Fang Han, Jianping Tang, Xuan Wang

**Affiliations:** ^1^ Department of Rheumatology and Immunology, Tongji Hospital, School of Medicine, Tongji University, Shanghai, China; ^2^ Center for Cancer Immunology, Faculty of Pharmaceutical Sciences, Shenzhen Institute of Advanced Technology (SIAT), Chinese Academy of Sciences (CAS), Shenzhen, China; ^3^ Department of Radiology, Tongji Hospital, School of Medicine, Tongji University, Shanghai, China

**Keywords:** Sjögrens syndrome (SjS), DNA methylation, epigenetics, demethylation, autoimmunity, systemic autoimmune disease, T cells

## Abstract

Sjögren’s syndrome (SjS) is a systemic, highly diverse, and chronic autoimmune disease with a significant global prevalence. It is a complex condition that requires careful management and monitoring. Recent research indicates that epigenetic mechanisms contribute to the pathophysiology of SjS by modulating gene expression and genome stability. DNA methylation, a form of epigenetic modification, is the fundamental mechanism that modifies the expression of various genes by modifying the transcriptional availability of regulatory regions within the genome. In general, adding a methyl group to DNA is linked with the inhibition of genes because it changes the chromatin structure. DNA methylation changes the fate of multiple immune cells, such as it leads to the transition of naïve lymphocytes to effector lymphocytes. A lack of central epigenetic enzymes frequently results in abnormal immune activation. Alterations in epigenetic modifications within immune cells or salivary gland epithelial cells are frequently detected during the pathogenesis of SjS, representing a robust association with autoimmune responses. The analysis of genome methylation is a beneficial tool for establishing connections between epigenetic changes within different cell types and their association with SjS. In various studies related to SjS, most differentially methylated regions are in the human leukocyte antigen (HLA) locus. Notably, the demethylation of various sites in the genome is often observed in SjS patients. The most strongly linked differentially methylated regions in SjS patients are found within genes regulated by type I interferon. This demethylation process is partly related to B-cell infiltration and disease progression. In addition, DNA demethylation of the runt-related transcription factor (RUNX1) gene, lymphotoxin-α (LTA), and myxovirus resistance protein A (MxA) is associated with SjS. It may assist the early diagnosis of SjS by serving as a potential biomarker. Therefore, this review offers a detailed insight into the function of DNA methylation in SjS and helps researchers to identify potential biomarkers in diagnosis, prognosis, and therapeutic targets.

## Introduction

Sjögren’s syndrome (SjS) is a chronic systemic autoimmune disease distressing a broad range (0.01% to 0.72%) of the general population (1). It predominantly distresses middle-aged individuals, particularly females, in their 40’s to 60’s (2). The symptoms of SjS evolve from simple dryness in the mouth and eyes to systemic, ultimately leading to lymphoma development (3). There is no radical cure for SjS, as the pathogenesis of SjS is not yet clear. The clinical spectrum is primarily characterized by sicca syndrome, resulting from immune-regulated glandular involvement. Besides that, musculoskeletal pain, fatigue, and systemic symptoms are present in a noteworthy fraction of diseased individuals. Lymphoma, however, presents as a complication in approximately 2%-5% of patients (4, 5). Significant features of the immunopathogenesis of SjS embrace increased B-cell activity, B-cell-T-cell interactions leading to ectopic lymphoid tissue formation, salivary epithelial cell dysfunction with increased apoptosis, autoantigen presentation, sustained elevation of proinflammatory cytokines, and increased level of genes regulated by type I interferon (6). Although the origin of SjS has not yet been completely clarified, it has been understood that environmental, genetic, and epigenetic factors significantly contribute to the progression of SjS, leading to the deregulation of epithelial cell function, inflammation, and autoimmune responses (7).

Genome-wide association studies (GWAS) confirm that genetic variations have been found to be associated with developing SjS in different populations. Genetic factors linked to SjS comprise human leukocyte antigen (HLA) allele subtypes and certain gene polymorphisms (8). However, these mutations only account for a small proportion of susceptibility to SjS. Although environmental factors, such as infections, e.g., the Epstein–Barr virus (EBV) infection (9), are considerably linked to the pathogenesis of SjS, no association was established between the reactivation phase of EBV and the onset of SjS symptoms (9). The specific roles of environmental factors, immunity, lifestyle, and genetics in the pathogenesis of SjS remain unclear. Thus, epigenetic mechanisms, for instance, non-coding RNAs, histone modifications, and DNA methylation, may act as a dynamic bridge between phenotypic expression, genome, and the environment through their regulatory impacts on the expression of genes (10).

A growing number of studies have revealed altered epigenetic landscapes in SjS, suggesting that epigenetic mechanisms contribute to SjS (3, 11–15). Studies in SjS have demonstrated the importance of DNA methylation patterns in the onset of disease and illuminated their role in the pathogenic behavior of various immune cells, cell-specific signaling pathways, and the activation/repression of downstream transcription factors (16, 17). As the cause of SjS is currently unknown and its development is multifaceted, there is still an absence of optimal drugs and treatment approaches. Recently, studies into DNA methylation within the context of SjS have observed a significant surge. This imitates the pivotal function of DNA methylation in the pathogenesis and progression of SjS. Cumulative evidence suggests an increasing focus on unraveling the complex interplay between DNA methylation patterns and the molecular mechanisms underlying SjS, thus identifying its potential as a critical regulator in SjS etiology. This review provides a significant understanding of the basic mechanics of SjS concerning DNA methylation and offers a promising avenue for identifying novel diagnostic, prognostic, and therapeutic targets.

## Mechanism of DNA methylation and DNA demethylation

**Figure 1:**
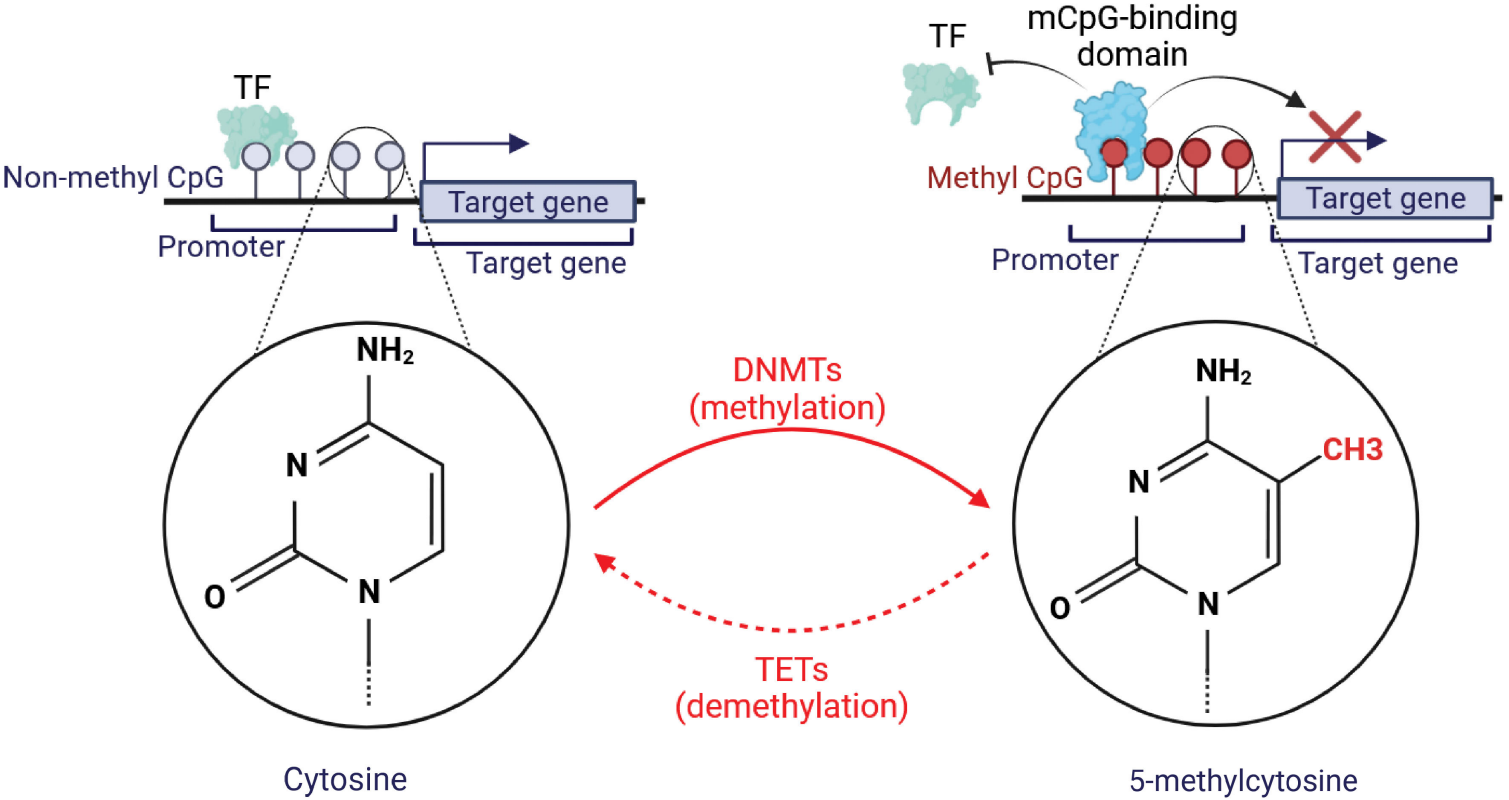
Graphical illustration of DNA methylation and its basic mechanism. The formation of novel DNA methylation patterns is modulated by methyltransferases (DNMTs). DNMTs aid in the formation of 5-methylcytosine from cytosine. This was followed by binding a methyl CpG-binding protein to methylated CpG sequences, which limits the ability of transcription factors (TFs) to reach this region, preventing the target genes’ transcription. Conversely, Ten Eleven Translocation (TET) proteins may initiate the demethylation and convert 5-methylcytosine to cytosine. This demethylation promotes the binding of TFs with non-methylated CpG to accelerate the expression of target genes.

DNA methylation is manifested as a pre-transcriptional epigenetic modification in the DNA, including methylation and demethylation. In the course of DNA methylation, DNMTs, via S-adenosyl-methionine (SAM), transfer a methyl group to the carbon-5 spot of the cytosine pyrimidine ring (5mC) within cytosine-guanine (CpG) dinucleotides (18). Methylation of CpG sites results in structural deviations of chromatin that impede transcription factor binding. CpG islands, with an abundance of CpG base pairs, are primarily located in the first exon regions and promoter regions of the genes. Therefore, DNA methylation of CpG islands in chromatin is typically regarded as an indicator of gene repression (14). The gene regulation through DNA methylation has been illustrated in **Figure 1**.

The regulation of the DNA methylation group is strictly and dynamically controlled by both ‘writers’ and ‘erasers’ who modulate epigenetic marks in opposite directions through enzymatic activity. Enzymes known as DNA methyltransferases (DNMTs) have been identified as a class of enzymes that not only maintain methylated sites following DNA replication (DNMT1) but also place new methylation sites (de novo methyltransferases: DNMT3A and DNMT3B) (19). The ten‐eleven translocation (TET) family and the DNA repair enzyme thymine DNA glycosylase (TDG) are the primary enzymes responsible for active DNA demethylation in mammals. Three mechanisms for demethylation have been identified: active demethylation (replication-independent), passive demethylation mediated by TET (replication-dependent), and 5mC deamination. In replication-independent active demethylation, TET dioxygenases oxidize 5mC to 5-hydroxymethylcytosine (5hmC), then to 5-formylcytosine (5fC), and finally to 5-carboxylcytosine (5caC) (20).

Previous research utilizing organisms lacking the DNMT1 enzyme demonstrated significant reductions in the methylation level of genomic DNA at CpG-rich repetitive regions and imprinted genes (21–23). In the deficiency of these enzymes, CpG-rich retroviral and intracisternal A particle (IAP) elements became slightly demethylated, while Igf-2 and Xist turned widely demethylated, according to recent studies using cells deficient in both the DNMT3A and -3B enzymes. This suggests that DNMT1 alone had sequence specificity for sustaining the methylation of these sequences (19).

Most 5mC in mammalian cells happens at DNA sequences involving CpG dinucleotides. Within human somatic cells, 70–80% of CpG sites are methylated, and the majority of unmethylated CpG sites are grouped on the CpG island, which is situated in the gene promoter region (24, 25). DNA methylation has been demonstrated to be crucial for several biological processes, such as X chromosome inactivation, genomic imprinting, chromosomal stability, and gene control (26). Numerous investigations have demonstrated that DNA methylation regulation is essential to mammalian developmental processes (27). A noteworthy example arises from the differentiation of stem cells. Hematopoietic stem cells (HSCs) give rise to all myeloid and lymphoid blood lineages (28). During this process, the methylation status of some genes (e.g., KCNH2, SUSD3) that determine cell destiny is highly controlled (29). On the other hand, several studies have demonstrated the connection between aberrant DNA methylation and the development of several human diseases (26, 30). This includes the activation of tumor promoter genes (such as MAGE and S100P) and the silencing of tumor suppressor genes (such as VHL and MLH1) in a variety of cancers (31, 32). Furthermore, studies have shown that aberrant methylation is a significant factor in the pathophysiology of neurological disorders like autism spectrum disorder, metabolic syndromes like hyperglycemia, and autoimmune diseases like idiopathic human lupus (33–35).

In broad terms, the inhibition of gene expression is tightly linked to DNA methylation in regions adjacent to the transcription start site (TSS) (36, 37). Based on accumulating data, transcriptional suppression of DNA methylation entails either the recruitment of transcription inhibiting factor (MeCP2) or the prevention of transcription activation factor (AP-2) from binding to TSS regions (38–40). Conversely, studies have suggested that increased transcriptional activity may result from gene body methylation (41, 42). Recent research has demonstrated that by ensuring the integrity of mRNA transcription beginning, DNA methylation on gene functions can shield the gene from spurious transcripts (43).

## DNA methylation in autoimmune disease

As a crucial regulator in the development and differentiation of immune cells, impaired DNA methylation profiles may substantially induce autoreactivity in the immune cells. This predisposes a person to immune irregularities and increases the possibility of progression in autoimmune anomalies (44–46). Epigenetic modification via DNA methylation recently gained consideration as a potential biomarker (47, 48). Several genes sensitive to DNA methylation and are linked with systemic lupus erythematosus (SLE) and other autoimmune illnesses were discovered to be hypomethylated in lymphocytes, especially CD4^+^ T cells. These genes include perforin (PRF1), integrin alpha L (ITGAL/CD11a), and lymphocyte function-associated antigen-1 (LFA1), which exhibit indistinguishable characteristics from CD8^+^ T cells (48–50). It is evident that DNA methylation alteration exhibits a constructive correlation with the activity of autoimmune diseases (51). For instance, MX dynamin-like GTPase 1 (MX1) and type I interferon (IFN) are suggested as latent biomarkers for the activity of SjS and SLE diseases (51, 52), and *IFI44L* is also referred to as a signatory gene of the type I IFN signaling (53). Moreover, the promoter methylation of interferon-induced protein 44-like (*IFI44L*) is a biomarker that can be found in the blood and can be used to monitor changes in the activity of SLE (54). Several investigations have exhibited that RA patients have an altered DNA methylation pattern in T cells, monocytes, B cells, synovial fibroblasts, and PBMCs (55–57). Similar to this, hypomethylated areas in the promoters of the enzymes dual specificity phosphatase 22 *(DUSP22)* and cytochrome P450 2E1 *(CYP2E1)* were linked to erosive and active disease as observed in peripheral blood specimens from patients with RA (58). Less research has been done on DNA methylation’s role in spondyloarthropathies (SpA) compared to RA (59, 60). Ankylosing spondylitis (AS) patients’ peripheral blood has been found to have altered DNA methylation levels (61–63). In AS patients but not in healthy controls, the inflammatory gene SOCS-1 (suppressor of cytokine signaling 1) is methylated (64). Accumulating evidence suggests that DNA methylation contributes to autoimmune disease through various immune cells, cellular signaling, and the modifications of the downstream transcriptional factors (54, 56, 58, 65–70). The function of DNA methylation in various autoimmune diseases is listed in **Table 1** (54, 56, 58, 65, 67, 69–71). In conclusion, a thorough knowledge of the role of DNA methylation by characterizing its modifications and identifying approaches to modify and achieve the desired level and course of anti-autoimmune responses may provide possible strategies for improved monitoring, diagnosis, and mitigation of disease progression driven by epigenetic modifications.

**Table 1:** Compilation of studies regarding autoimmune rheumatic diseases and DNA methylation in cells.

Reference	Study design for DNA methylation	Cell types	Main outcomes for DNA methylation
Lu Q, et al. (65)	Bisulfite sequencing was used to determine the methylation status of the *ITGAL* promoter and flanking regions in T cells from lupus patients and healthy subjects.	T cells in SLE	Bisulfite sequencing was used to determine the methylation status of the *ITGAL* promoter and flanking regions in T cells from lupus patients and healthy subjects and in T cells treated with DNA methylation inhibitors.
Lu Q, et al. (71)	Decreased global DNA methylation, T cell DNA was isolated, and bisulfite treated using published protocols, then the promoter and enhancer were amplified using nested primers to determine whether *CD40LG* on the inactive X demethylates and is overexpressed uniquely in women with lupus	T cells in SLE	promoter and enhancer demethylation may cause *CD40LG* overexpression on CD4^+^ T cells in women but not men with lupus.
Miller S, et al. (67)	Genome-wide DNA methylation of lupus and age, sex, and ethnicity-matched control CD8^+^ T cells was measured using the Infinium Methylation EPIC arrays	CD8^+^ T cells in SLE	55% of genes had hypermethylated CpG sites, 38% had hypomethylated CpG sites, and the remaining 7% of genes exhibited a mixed methylation pattern at CpG sites in CD19^+^ B cells. Genes with significantly different DNA methylation patterns are involved in functional pathways required for B cell signaling, inflammation and autoreactivity.
Zhao M, et al. (54)	*IFI44L* promoter methylation was examined using DNA samples from a discovery set including 377 patients with SLE, 358 healthy controls and 353 patients with RA	Whole blood cells in SLE	The methylation levels of Site1 and Site2 within the *IFI44L* promoter were significantly lower in patients with SLE with renal damage than those without renal damage. Patients with SLE showed significantly increased methylation levels of Site1 and Site2 during remission compared with the active stage.
Sun X, et al. (69)	Bisulfite sequencing was performed to determine the methylation status of the *FOXP3* proximal promoter sequences	CD4^+^ T cells in systemic sclerosis	in CD4^+^ T cells from patients with systemic sclerosis, treatment with all-*trans* retinoic acid, a natural derivative of vitamin A, increases the expression of *FOXP3* and, subsequently the proportion of T_reg_ cells by promoting demethylation of the *FOXP3* promoter.
Julià A, et al. (70)	CpG methylation in isolated B lymphocytes was assayed on the Illumina HumanMethylation450 BeadChip in a discovery cohort of RA patients (N =50) and controls (N=75). Differential methylation was observed in 64 CpG sites	B cells in RA	in CD19^+^ B cells, many relevant genes are differentially methylated in patients with RA compared to healthy individuals. These genes include *CD1C*, *TNFSF10*, *PARVG*, *NID1*, *DHRS12*, *ITPK1*, *ACSF3* and *TNFRSF13C*, all of which were identified in a discovery cohort of patients with RA and validated in an independent cohort.
Mok A, et al. (58)	Fluorescence-activated cell sorting was used to separate the cells into 4 immune cell subpopulations (CD14^+^ monocytes, CD19^+^ B cells, CD4^+^ naive T cells, and CD4^+^ memory T cells), and 229 epigenome-wide DNA methylation profiles were generated using Illumina HumanMethylation450 BeadChips	CD14^+^ monocytes, CD19^+^ B cells, CD4^+^ naive T cells, and CD4^+^ memory T cells in RA	Differential methylation of CpG sites associated with clinical outcomes was observed in all 4 cell types. Hypomethylated regions in the *CYP2E1* and *DUSP22* gene promoters were associated with active and erosive disease, respectively.
Rodríguez-UbrevaJ, et al. (56)	High-throughput DNA methylation analyses of patients with RA and controls and in vitro cytokine stimulation were used to investigate the underlying mechanisms behind DNA methylation alterations in RA as well as their relationship with clinical parameters, including RA disease activity	peripheral blood monocytes in RA	The DNA methylomes of peripheral blood monocytes displayed significant changes and increased variability in patients with RA with respect to healthy controls. Changes in the monocyte methylome correlate with DAS28.

SLE, systemic lupus erythematosus; ITGAL, lymphocyte function-associated antigen-1 (LFA1), integrin alpha L; CpG sites, cytosine guanine dinucleotide (CpG) sites; IFI44L, interferon-induced protein 44-like; FOXP3, forkhead box protein 3; RA, Rheumatoid arthritis; CYP2E1, cytochrome P450 2E1; DUSP22, dual specificity phosphatase 22; DAS28, Disease Activity Score in 28 joints.

## Role of DNA methylation in SjS

DNA methylation is a reflection of the epigenetic status at a particular point that can impact the activity of disease through the modification of gene-level and downstream pathways (72). By engaging proteins entailed in gene repression or by preventing transcription factors from binding to DNA, DNA methylation controls gene expression (48, 73, 74). An EWAS examined the association of European League Against Rheumatism (EULAR) Sjögren Syndrome Disease Activity Index (ESSDAI) score with DNA methylation and discovered that patients with high ESSDAI had significantly more differentially methylated regions than those with low ESSDAI (75). The etiology of chronic fatigue, a leading reason for disability in patients with SjS, is not well stated. Norheim and colleagues performed an epigenome-wide DNA methylation patterns analysis to examine the possible involvement of DNA methylation in fatigue in SjS. The outcomes revealed 251 CpG sites with differential methylation, with the main finding being hypomethylation of a non-coding RNA in high fatigue (76). When compared to other epigenetic alterations, DNA methylation has a greater degree of stability, making it an effective marker for use as a diagnostic indicator (77). Over the recent years, several research teams have demonstrated that a range of changes in DNA methylation patterns are linked to autoimmune rheumatic diseases. These alterations have been found to be associated with different subtypes of these diseases, as well as with their activity levels and overall severity.

*Lymphotoxin-α* (*LTA*), belonging to the TNF superfamily and secreted by CD8^+^, Th17, B cells, and NK, is recognized for its pro-inflammatory properties (78). Mechanistically, *LTA* operates through two distinct signaling routes. Firstly, as an *LTA* trimer, it binds to TNF1 and TNF2 receptors, driving lymphangion genesis and elevating the secretion of chemokines and cytokines (79–82). Secondly, it competes with *lymphotoxin-β (LTB)* to shape a trimeric ligand that binds to the *LTB* receptor on lymphoid cells and stimulates various pathways, such as NF-KB (83–85). The hypomethylation at numerous regions of *lymphotoxin-α (LTA)* has been found (81, 86). It is interesting to note that genetic variations in LTA are linked with vulnerability to SjS, as *LTA* has been found in the salivary glands of SjS patients (87, 88), thereby acting as a critical factor in the progression of SjS (89). It was estimated that deletion of *LTA* in IL-14α transgenic mice resulted in normal salivary gland secretion rate and no lymphocytic infiltration (89). A recent work by Altorok et al. (52) employing genome-wide DNA methylation has shown that naïve T cells of pSjS had hypomethylated *LTA* gene. Additionally, pSjS and several SNP in the *LTA/LTB/TNFα* locus have been shown to strongly correlate as found by single-nucleotide polymorphism (SNP) analysis (87).

Meanwhile, *IFI44L* (interferon-induced protein 44-like) promoter undergoes DNA methylation in various autoimmune diseases (51, 90). Also, *IFI44L* is a recently discovered gene that has been implicated in predisposing people to certain infectious diseases. The analysis of genome-wide DNA methylation patterns identified hypomethylation of I*FI44L* in whole blood in SjS as the most significant finding in these two large-scale studies. *IFI44L* has been identified as a signature gene for the IFN-I pathway in SjS (52, 53, 75). It was estimated that methylation of the *IFI44L* promoter could accurately differentiate between patients with SLE and healthy individuals with a high degree of sensitivity and specificity (54). However, a recent study used the microarray and sc-RNA analysis and concluded that *IFI44L* is a highly expressed shared gene in SLE and SjS (91). Additionally, several genes, including *BLK* (B lymphoid tyrosine kinase), *STAT4* (signal transducer and activator of transcription 4), *CXCR5* (C-X-C chemokine receptor type 5), *IL12A* (interleukin-12 subunit alpha), *TNIP1* (TNFAIP3-interacting protein 1), and *IRF5* (interferon regulatory factor 5), have been suggested as potential gene candidates for susceptibility to SjS in various studies (92). The pathophysiology underlying SjS is complex. Thus, only a few studies have explored the attribution of DNA methylation in SjS. It has been reported that the most frequent modification observed in the genome of SjS patients is the demethylation of several sites (6, 93, 94).

Type I interferon (IFN-I) is known to perform a crucial function in the pathophysiology of autoimmune diseases, particularly in the progression of SjS (95, 96). Interestingly, studies indicate that the most strongly linked differentially methylated positions and regions in SjS patients are situated within genes regulated by type I interferon (13). Interferon-activated Myxovirus-resistance proteins (Mx) are an excellent tool for assessing IFN-I in autoimmune disorders (97), especially distinguishing the SjS using serum and saliva (98). Among various Mx proteins, MxA is the most applicable and suitable biomarker for SjS (99). MxA levels are linked to signs of active disease and decrease in patients treated with hydroxychloroquine, indicating the potential clinical usefulness of MxA in categorizing patients based on IFN positivity (99, 100).Activation of the IFN-I pathway is observed as one of the vital pathways in the pathophysiology of SjS, and it is particularly pronounced in individuals who have antibodies to SSA/SSB (101, 102). However, the connection between IFN-regulated genes and DNA methylation is complex and multifaceted, especially in SjS. Both SLE and SjS patients have demonstrated significant cell-specific epigenome-wide and genomic-wide hypomethylation of IFN-regulated genes in the epithelial cells from minor salivary gland as well as multiple tissues, with numerous sites being linked to augmented levels of IFN-regulated genes (13, 100, 103). The interferon regulatory factor 5 (IRF5) gene, which encodes a transcription factor that contributes to the modulation of IFN-induced genes and synthesis of IFN-α, is the most important genetically susceptible locus for SjS irrespective of the HLA region (104, 105). An earlier study examined the differential methylation positions and regions in whole blood. It was found that IFN-I-regulated genes were enriched in the differentially methylated positions and regions with the strongest associations. Additionally, these areas were found to be improved in pathways associated with extracellular matrix assembly and collagen metabolism. Moreover, the identified epigenetic signatures were exclusively detected in patients who tested positive for anti-SSA/Ro antibodies (106). Likewise, another study identified significant genome-wide hypomethylation of IFN-modulated genes in B cells and whole blood (100).

Besides the importance of IFN-I in SjS, the calcium and Wnt pathways were identified as important regulatory molecules in the salivary gland epithelial cells (SGECs) in the course of SjS progression. Cell-specific epigenome-wide analysis showed that genes involved in these pathways are enriched for hypomethylation and hypermethylation at differentially methylated CpG sites (DMCs) (103). The alteration in the DNA methylation of critical pathways provides a theoretical basis for a therapeutic target in SjS (**Table 2**) (66, 68, 75, 76, 105, 107–110, 112).

**Table 2:** Compilation of studies regarding Sjögren’s syndrome and DNA methylation in cells.

Country Reference	Study design for DNA methylation	Cell types	Main outcomes for DNA methylation
Norway BrækkeNorheim K et al. (76)	Methylation analysis of patients in pSS patients with high or low fatigue	Whole blood	251 differentially methylated CpG sites were associated with fatigue. The CpG site with the most pronounced hypomethylation in pSS high fatigue annotated to the SBF2-antisense RNA1 gene. The most distinct hypermethylation was observed at a CpG site annotated to the lymphotoxin alpha gene.
France Thabet Y et al. (66)	Methylation analysis and transcript levels of DNMTs in patients with and without SS	Peripheral B and T cells; SGECs	Global demethylation and reduction in DNMT1 transcript levels in SGECs of SS patients. No differences in methylation profile for B and T cells.
France Gestermann N et al. (105)	Methylation analysis of IRF5 promoter region to determine if this could be the cause for increased IRF5 mRNA expression in patients with SS	Peripheral B and T cells	DNA methylation profile of T CD4+ and B lymphocytes did not differ between SS patients and controls.
France Miceli-Richard C et al. (75)	Methylation comparison in profiles in the CD4^+^ T cells and CD19^+^ B cells of pSS patients and controls	CD19^+^ B cells	55% of genes had hypermethylated CpG sites, 38% had hypomethylated CpG sites, and the remaining 7% of genes exhibited a mixed methylation pattern at CpG sites in CD19^+^ B cells. Genes with significantly different DNA methylation patterns are involved in functional pathways required for inflammation.
China Yu X et al. (68)	Methylation analysis of *FOXP3* promoter region to determine whether the *FOXP3* promoter is hypermethylated and whether aberrant *FOXP3* expression occurs in CD4^+^ T cells from patients with pSS	CD4^+^ T cells	Hypermethylation at the promoter of *FOXP3* in CD4^+^T cells of pSS patients. A decrease in expression in protein *FOXP3* mRNA and protein in CD4^+^T cells of pSS patients.
China Yin H et al. (107)	Methylation analysis of TNFSF7 promoter region	CD4^+^ T cells	Hypermethylation at the promoter of TNFSF7 in pSS CD4^+^T cells. Demethylation of the CD70 promoter regulatory elements contributes to CD70 overexpression in pSS CD4^+^ T cells and may contribute to autoreactivity.
France Belkhir R et al. (108)	methylation analysis of CD40L promoter region	CD4^+^ T cell	No significant differences in methylation profiles between patients and controls.
Greece Mavragani CP et al. (109)	Methylation analysis of LINE-1; expression analysis of DNMT1, DNMT3A, DNMT3B, MeCP2 in SS patients and controls	SGECs	↑ levels of DNMT1, DNMT3B and MeCP2 transcripts in SS patients.
France Konsta OD et al. (110)	Methylation analysis at SSB promoter region in patients with pSS and cell cultures	SGECs	↓ global DNA methylation levels in patients with SS. ↓ global methylation associated with lymphocyte infiltration in MiSG; ↓ global methylation associated with anti-La/SSB positive SS cases demethylation at SSB promoter caused higher levels of transcripts and SSB super expression in 5-Aza-C-treated HSG cells.
France Konsta OD et al. (111)	Methylation analysis of the KRT19 locus to investigate epigenetic regulation of expression in patients with SS	SGECs	↓ global DNA methylation in pSS patients is associated with demethylation of the KRT19 locus as well as with overexpression of the KRT19 protein.
USA Chi C et al. (112)	Methylation analysis in patients with and without SS	SGECs	215 DMRs in SS patients: 169 hypermethylated regions related to nervous system development, cell signaling and transport; and 46 hypomethylated regions related to immune function.
USA Cole MB et al. (113)	Methylation analysis in patients with varying phenotypes of SS	SGECs	7,820 DMPs associated with disease status (5,699 hypomethylated and 2,121 hypermethylated DMPs); 57 of the genes with DMPs are involved with the immune response; extensive hypomethylated region near genes PSMB8 and TAP1.
Chile Sepúlveda D et al. (114)	Methylation and expression analysis in genes of the IRE1α/XBP-1 pathway in SS and control patients	SGECs	Hypermethylation in IRE1α, XBP-1 and GRP78 promoter region and diminished transcript levels; ↓ protein levels for IRE1α, XBP-1s and GRP78 in SS patients.

SS, Sjögren’s syndrome; pSS, Primary Sjögren’s syndrome; SGECs, salivary gland epithelial cells; CpG sites, cytosine guanine dinucleotide (CpG) sites; DNMTs, DNA methyltransferase 1; MiSG, minor salivary gland; FOXP3, fork head Box Protein 3; LINE-1, long interspersed repeat element 1; anti, SSB, anti-Sjögren’s syndrome antigen B; HSG, human salivary gland; KRT19, keratin 19; DMRs, Differentially methylated regions; DMPs, differentially methylated positions; IRE1α, inositol-requiring enzyme 1alpha; XBP-1s, X-box binding protein 1s; GRP78, glucose-regulating protein 78.

Our understanding of the relationship between these pathways involved in DNA methylation and gene regulation is evolving, as epigenetic regulation is a multifaceted process prejudiced by various factors. Meanwhile, the interplay between these signaling pathways and DNA methylation is more likely depending on the context and is highly intricate. Further research is required to uncover more details about these interactions and their implications for SjS. Interestingly, besides providing novel therapeutic targets, using DNA methylation arrays in a clinical setting can also advance researchers understanding of classifying SjS phenotype (112).

### DNA methylation in various cells during SjS progression

**Figure 2:**
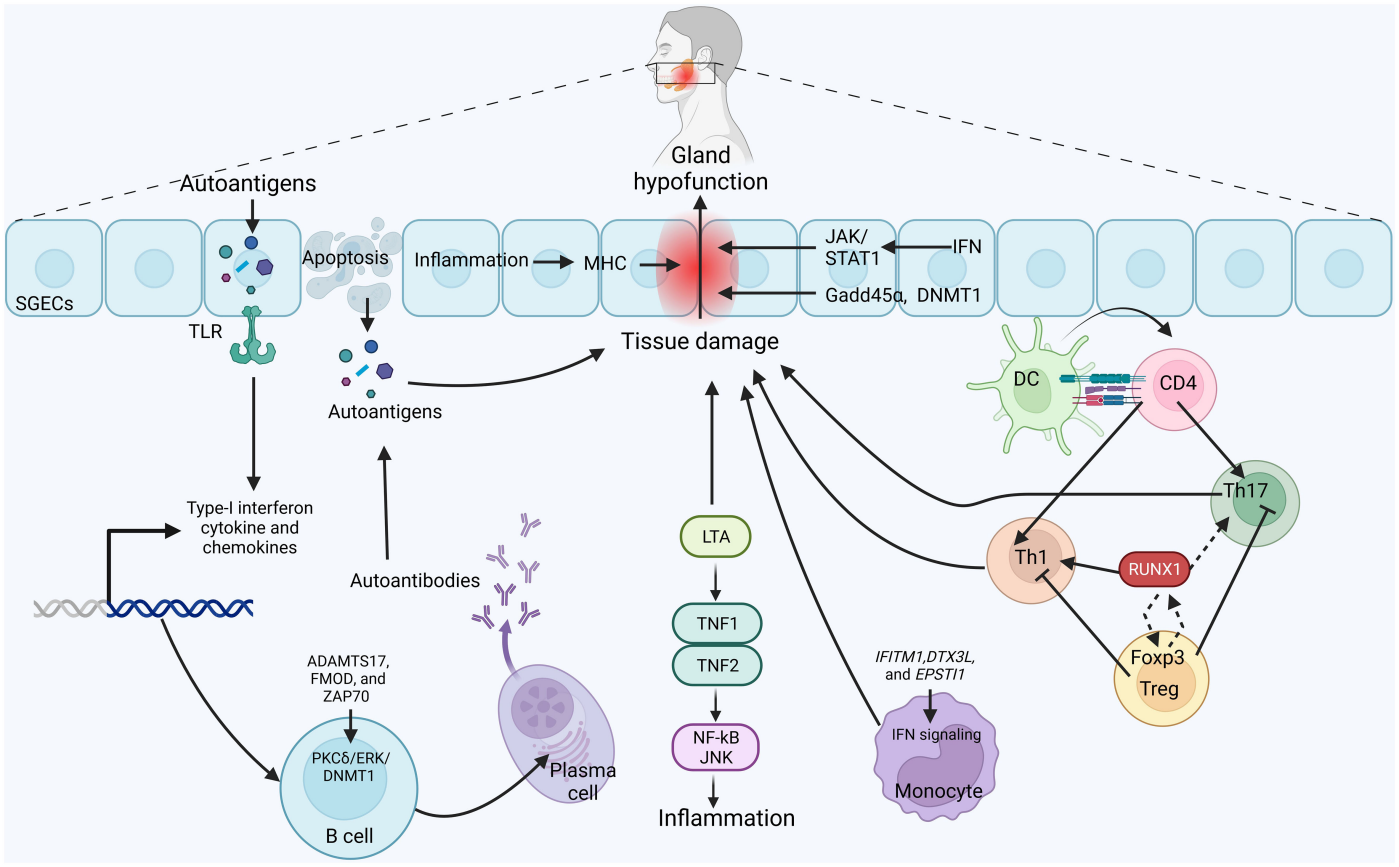
Possible mechanism of DNA-methylation in the pathogenesis of SjS: Activation of toll-like receptors (TLRs) signaling, predominantly activated in the salivary gland epithelial cells (SGECs), produces autoantigens which enhances the production of immunocompetent cytokines and chemokines and cytokines. These lead to the apoptosis of SGECs, epithelial hypofunction and tissue damage. Autoantigens can be released from SGECs and presented to immune cells. CD4+ T cells differentiate into inflammatory Th1 and Th17 to participate in the tissue damage. Immunosuppressive Tregs inhibit the activity of inflammatory Th1 and Th17 cells. Methylation of RUNX1 activates the Th1 cells, whereas elevated RUNX1 binds with Foxp3 in Tregs, leading to transcriptional modification and enhancing its expression, which in turn inhibits the function of RUNX1. Meanwhile, LTA binds with TNF1 and TH=NF2 to activate JNK/NF-kB signaling, leading to inflammation. In addition, DNA methylation pattern of ADAMTS17, FMOD and ZAP70 is altered in a PKCδ/ERK/DNMT1-dependent manner. Increased B cells also activate plasma cells to produce antibodies against autoantigens. Meanwhile, DNA methylation of IFITM1, DTX3L and EPSTI1 activates the IFN signaling in monocytes to alleviate inflammation and tissue damage. Strikingly, inflammation-induced DNA methylation regions in major histocompatibility complex, IFN signaling induced JAK/STAT1 signaling, and altered levels of Gadd45α level and DNMT1 in SGECs also participates in the pathogenesis of SjS.

The immune system involves various types of immune cells, each with specific functions for the progression of autoimmune diseases (115). In the context of SjS, immune cells, particularly lymphocytes, infiltrate the affected glands and contribute to inflammation and tissue damage. These immune cells release cytokines and other signaling molecules that further promote inflammation and disrupt normal gland function (116, 117). The interaction between SjS and immune cells is complex. While attempting to regulate the autoimmune response, immune cells can inadvertently contribute to the damage to glandular tissues. Additionally, the chronic inflammation associated with SjS can have systemic effects beyond the glands, impacting various organs and tissues throughout the body (7, 118). Recent studies show that DNA methylation is applied to many cell types implicated in the pathophysiology of SjS. Among these, SGECs, lymphocytes, and monocytes (17, 88) are significant factors contributing to the SjS progression after being subjected to DNA methylation. As evidence increases, these differential DNA methylation genes in immune and non-immune cells may be used as candidate biomarkers to predict SjS. Here, we have discussed the importance of DNA methylation in these cell types and how it plays an imperative function in SjS (**Figure 2**).

### DNA methylation in T cells

T cells, including CD4^+^ and CD8^+^ T cells, are overwhelming infiltrators in most phases of the SjS (116). Specifically, the engagement of various T-cell subsets in Sjögren’s syndrome (SjS) underscores the remarkable complexity of the disease’s pathogenesis (116). It was determined that in SjS, T cell activation results in tissue damage, B cell activation, inflammatory cell infiltration, and metabolic alterations (119). DNA methylation is crucial for the differentiation of T cells (120, 121). In the process of becoming activated and differentiating from naive CD4^+^ and CD8^+^ T cells to effector cells, there are alterations in the DNA methylation patterns of the promoters associated with effector genes, such as Il2, Tnfa, and Ifng. These changes in DNA methylation may exhibit a progressive decline throughout differentiation (122). The initial Epigenome-Wide Association Study (EWAS) in SjS focused on examining DNA methylation in immature CD4^+^CD45RA^+^ T cells isolated from 11 female SjS individuals and 11 matching controls. The authors found that there was hypomethylation of multiple sites of *LTA* and IFN-regulated genes, including interferon-induced transmembrane protein 1 *(IFITM1)*, activators of transcription 1 *(STAT1)*, and *IFI44L* (52). Similarly, in another study, four hypomethylated *STAT1*, homo sapiens SH3 and PX domains 2A (*SH3PXD2A*), friend leukemia virus integration (*FLI)37453*, ubiquitin-specific peptidase 18 *(USP18)* and one hypermethylated gene F-box and leucine-rich repeat protein 16 *(FBXL16)* were found in T cells (75). Likewise, Yin et al. found that CD70 overexpression results from demethylation of the CD70 promoter regulatory regions in SjS CD4+ T cells, which may be a factor in their autoreactivity (107).

Among different subsets of CD4^+^ T cells, regulatory T cells (Tregs) are fundamental in maintaining normal physiology during the progression of SjS (123). The stability and role of Foxp3^+^ Tregs is highly reliant on DNA methylation (124). It was suggested that reduced expression of *FOXP3* in CD4^+^ T cells in SjS is associated with DNA hypermethylation (68). RUNX1 has been known to perform essential roles in developing the granular convoluted tubules (125). It was studied that *RUNX1* is tangled in the membrane trafficking of particular proteins of the acinar cells in the submandibular gland, which further permits the proper secretion of saliva (126). Taking into account the function of *RUNX1* in saliva secretion, Altorok and colleagues have reported hypermethylation of the transcriptional factor RUNX1 in SjS individuals (52), which acts on regulating the development of HSCs (hematopoietic stem cells). The CNS2 region of Foxp3 has CpG islands, which undergo differential methylation. Conversely, Runx1, along with Cbfβ, demethylates Foxp3 by directly binding with the CNS2 element of Foxp3 (127, 128), thereby regulating the Foxp3 transcription by altering its chromatin structure (129). Meanwhile, RUNX1 expression has been linked to a susceptibility to cancer, implying a potential link to a predisposition to lymphoma in SjS patients (130). Collectively, RUNX1 enhances Foxp3, which in turn inhibits the RUNX1 activity and transforms RUNX1 from an activator into a repressor, or Foxp3 also contributes to Runx in activating or repressing its downstream target genes (129). Additional research is required to clarify the epigenetic processes underlying the pathophysiology of SjS since the function of DNA methylation in T cells is still unclear. These investigations have the possibility of significantly improving our understanding of SjS and establishing the groundwork for innovative, tailored therapeutic interventions.

### DNA methylation in B cells

A multistep mechanism leads to the overactivation of B lymphocytes, which is crucial in the pathophysiology of SjS (117). Salivary epithelial cells, the targets for SjS, continue to interact with subpopulations of B cells, which in return help to activate an autoimmune reaction in tissues by producing autoantibodies and consequently form immune complexes (131). It has been reviewed that aberrant expression of translational factors and modifications in the epigenetics in B cells is highly correlated with anomalous B cell functions in multiple diseases, including autoimmune diseases (132). For instance, analysis of differentially expressed and methylated genes shows the alterations of expression patterns and DNA methylation patterns of *ADAMTS17*, *FMOD*, and Z*AP70* in chronic lymphocytic leukemia (CLL) (133). They found that *ADAMTS17* was hypermethylated in the gene promoter region and hypomethylated in the gene body region. In contrast, *FMOD* and *ZAP70* were hypomethylated in the promoter region (133). Regarding the involvement of B cells in SjS, abundant genes with differential DNA methylation in genetic at-risk loci (*HLA-DRA, HLA-DQB1*, *IRF5*) were observed (66, 75). Methylation changes in B cells were common in patients who were positive for autoantibodies in a number of particular pathways, including IFN-modulated genes (75). Miceli-Richard and colleagues conducted an analysis of genome-wide methylation patterns in two distinct immune cell populations, namely peripheral CD4+ T cells and B cells, within a cohort of 26 women diagnosed with SjS and 22 control subjects with same age. Their study revealed more significant differences in DNA methylation patterns among B cells, as opposed to T cells, when comparing patients with SjS to the control group (75). Meanwhile, in an investigation by Altorok et al., only 119 differentially methylated CpG sites (DMCs) were found in CD4^+^ T cells, which is interesting because they used a lower cutoff level of 0.07 to detect differential methylation between patients and controls. On the other hand, B cells showed a strikingly larger number of 6,707 DMCs, impacting 3,619 genes. In these DMCs, SjS patients had hypomethylation at 44% of the differentially methylated locations, as revealed by genome-wide DNA methylation patterns. Notably, several of the genes activated by IFN were connected with certain hypomethylated CpG sites in B cells, indicating a potential connection between DNA methylation alterations and immunological activation in these cells (52). Imgenberg-KreuzJ et al. conducted a deeper look at the methylation of the whole genome in peripheral B lymphocytes. They determined that 5623 distinct genes had different levels of methylation, with the majority of these hypomethylated regions being assigned to genes that participate in immune response pathways, particularly IFN-regulated *MX1*, *IFI44L*, *poly* (*ADP-ribose*) *polymerase 9 (PARP9)* and *IFITM1* (100). Moreover, Thabet Y et al. conducted a study to analyze the global DNA methylation in the salivary gland epithelial cells (SGEC) and peripheral B and T cells from SS patients. They found that the overall methylation was decreased in SGECs. Surprisingly, by co-culturing human salivary gland cells and B cells, authors have found that SGEC demethylation may be caused in part by invading B cells, as suspected in patients treated with anti-CD20 antibodies to reduce B cells (66). Mechanistically, it was suggested that DNA demethylation mediated by B lymphocytes could be due to changes in the PKCδ/ERK/DNMT1 pathway as using rottlerin, PD98059 and 5-azacytidine to inhibit PKCδ/ERK/DNMT1 signaling reduces global DNA methylation in SGECs; however, when patients get the anti-B cell mAb rituximab, this process can be reversed (66, 111). All these findings suggest that anomalous B cell activation and cytokine secretion contribute significantly to the immunopathogenesis of SjS, and investigating the abnormal DNA methylation changes in B cells may highlight the potential for DNA methylation-targeted therapies to modulate the core anomalies driving the disease.

### DNA methylation in monocytes

There is evidence to suggest that the monocyte is a crucial actor linking multiple immune responses (134), as it performs a major role in the SjS (135). Over previous years, interest has grown in the impact of monocytic cells in the development of SjS. There is evidence that IFN-signaling and viral infection-related pathways are highly upregulated in monocytes involved in the pathogenesis of SjS (136). Like B and T cells, the function and biology of monocytes are also influenced by DNA methylation, predominantly in autoimmune diseases (134, 137). In circulating monocytes from individuals with SjS, DNA methylation alterations appear primarily as hypomethylation. Hypomethylation in *IFITM1, myxoma resistance1, PARP9, deltex E3 ubiquitin ligase 3L* (*DTX3L*), and epithelial-stromal interaction 1 (*EPSTI1)*, which eventually effect the IFN signaling in SjS monocytes, was testified. In patients with SSA/SSB autoantibodies, differently methylated genes were present in the ribosome and involved in AMP-activated protein kinase (AMPK) signaling pathway (138). Thus, changes in methylation may have an effect on IgG production through the influence of monocyte differentiation (139). These discoveries underscore the significance of gene regulation by DNA methylation in the dysfunctional classical monocytes across SjS patients.

## Association of salivary gland epithelial cells with DNA methylation

Aberrant DNA methylation has also been implicated in non-immune cells. Considering that SGECs play an essential function in the emergence of SjS (140), it has been found that SGECs also play a significant function in the immune regulation in the pathophysiology of SjS and influencing the initiation and continuation of autoimmune response and inflammation (7, 141, 142). As SjS advances, the fundamental variations in the proteome of SGECs between SjS and healthy controls provide tangible evidence of SGEC transformation into an innate immune cell (143). This shift is coupled with a notable redirection of cellular metabolism. These metabolic shifts primarily center around mitochondrial processes, also reflected in the structural changes observed within the cells (143). Conversely, IFN-γ-meditated ferroptosis of SGEC exacerbates SjS pathogenesis through JAK/STAT1, signifying the function of ferroptosis in SGECs in SjS-associated immunogenicity and inflammatory responses (144). Disparity in DNA methylation in salivary gland tissues can exacerbate the progression of autoimmune diseases (50, 112). Minor salivary gland-based epigenome-wide DNA methylation found a decreased global DNA methylation in the SGECs from SjS patients. SGEC demethylation in SjS patients was linked with a 2-fold upsurge in *Gadd45α* level and a 7-fold reduction in DNMT1 (66). A genome-wide methylation investigation was conducted by Cole et al. using minor salivary glands from 13 SjS patients and 13 control participants. In SjS, a study utilized genome-wide DNA methylation analysis on the human labial salivary gland biopsy specimens and discovered 7820 sites had variable methylation, of which 5699 had hypomethylation, and 2121 had hypermethylation (113). Likewise, genome-wide DNA methylation analysis conducted by Imgenberg-Kreuz et al. found 45 differentially methylation locations in minor salivary gland samples from 15 SjS individuals and 13 controls, with the IFN-induced gene *OAS2* having the most substantially hypomethylated site (100). Similarly, Konsta et al. (2016) showed a potential correlation between abridged DNA methylation in minor salivary glands and up-regulation of the KRT19 (keratin-19) in glandular acini. In a following investigation, the incubation of a human salivary gland cell line with the DNMT antagonist 5-azacytidine led to the amplification of the mRNA level of KRT19 and protein level of cytokeratin-19 (110, 111, 145). Moreover, another study thoroughly performed epigenomic-wide association study and analyzed 131 samples of labial salivary glands (LSGs) and illustrated that the major histocompatibility complex (MHC) region has a large number of DMRs, which are hypomethylated in genomic regions implicated in the immunological response in LSGs. To address the challenges posed by cellular heterogeneity, Charras and colleagues conducted their study using long-term cultured SGECs that were obtained from minor salivary glands from 8 individuals diagnosed with SjS and 4 control subjects. It is noteworthy that 2650 genes had 4662 differential methylation sites, among which 21% exhibited hypomethylated in SGECs from SjS. Interestingly, the data attained from these SGECs was in accordance with the data from whole minor salivary glands, as IFN-regulated genes were postulated as differentially methylated genes (103). HLA region constitutes almost 50% of the altered methylated regions, with the matching methylation quantitative trait loci (meQTLs) in the regions encircling the HLA-DQA2, HLA-DQB1, and HLA-DQA1 loci (146). This research identified unusual DNA methylation changes in SGECs. It highlighted the prospect role of HLA class DNA methylation modifications and other major pathways and genes in the pathophysiology of SjS. Non-immune cell epigenetic changes have been found to be more similar to autoimmune disease-induced inflammatory responses rather than being directly related to SjS (14). It has been suggested that these epigenetic changes may be part of the pathogenesis of SjS, but are not the direct cause (14). It is necessary to consider multiple factors, including autoimmune reactions, inflammatory responses, and non-immune cell epigenetic changes, for the diagnosis and treatment of SjS. Currently, the role of non-immune cell epigenetic changes in SjS is still unknown, which requires further research and exploration.

## Targeting DNA methylation as a potent therapeutic approach in SjS

SjS has no cure at present. The clinical treatment approaches currently in use and the available biomarkers can barely halt the advancement of SjS and cannot entirely anticipate how the disease will progress. To meet clinical needs, new biomarkers and molecular targets are instantly desirable. Many clinical trials for SjS-related drugs have failed to meet the primary endpoint due to unclear SjS assessment criteria, making it difficult to determine the extent to which the symptoms reflect underlying pathological biology (147). The emergence of epigenetic regulation, particularly DNA methylation, offers new insights into the treatment of SjS. Drugs related to DNA methylation mainly include traditional DNA hypomethylating agents (HMAs), such as decitabine (DAC) and azacitidine (AZA) (148). Even though epigenetics is a relatively new discipline that gradually emerged in the 1980s, HMAs were only approved in the 21st century for treating hematopoietic system tumors (149). Currently, there is a lack of evidence for the use of passive demethylation agents in SjS treatment.

In the last five years, much consideration has been given to Treg cells in DNA methylation studies of autoimmune diseases. Studies on other autoimmune rheumatic diseases have also demonstrated a correlation between autoimmunity and reduced *FOXP3* promoter DNA methylation. *FOXP3* promoter hypermethylation leads to reduced levels of *FOXP3* in CD4^+^ lymphocytes in SjS (68). In CD4^+^ lymphocytes derived from individuals afflicted with Systemic Sclerosis (SSc), the application of a naturally occurring vitamin A derivative (all-trans retinoic acid) upregulates *FOXP3* expression and, consequently, elevates the population of Tregs by inducing *FOXP3* promoter demethylation (69), which may act as a target for treatment in SjS.

Aberrant DNA methylation in B lymphocytes implies their role in the pathogenesis of SjS (100). B cells significantly contribute to most autoimmune conditions, as seen by the prevalence of autoantibodies in autoimmune rheumatic disorders and the efficiency of B cell-depleting therapy in certain conditions (150). The severity of SjS and B cell infiltration has been found to be negatively associated with the DNA methylation levels in SGECs. Furthermore, administering the anti-CD20 monoclonal antibody rituximab has been testified to increase the DNA methylation levels in the SGECs of SjS patients (151). It has been proven that a number of genes transcribed by SGECs, including B-cell activating factor (BAFF), aquaporin-1/5, and IFN pathway, are susceptible to the effects of rituximab (152, 153). Anti-B cell treatments that indirectly restore DNA methylation in SGECs bring up new therapeutic possibilities for SjS (154).

The activation of the IFN-I system is widely considered a crucial mechanism in the pathophysiology of SjS. Once elevated levels of IFN-α and downstream activation of interferon-stimulated genes (ISGs) were discovered in patients with SjS, strategies targeting IFN-I were promptly developed. Although not specific to hindering type I IFN signaling, various small molecule kinase inhibitors that are targeted at Janus Kinases (JAKs) are being trialed clinically for SjS, such as filgotinib (JAK1 inhibition) and lanraplenib (formerly GS-9876, Tyk2 inhibition) (94, 114, 145).

## Conclusion and perspectives

Recent genetic and epigenetic research into SjS has unveiled several underlying genes responsible for the disease, with immune cells playing a crucial role in its development. These important genetic and epigenetic discoveries have the potential to address several clinical needs, including improved diagnosis, patient classification, predictive indicators of associated diseases, for instance, heart disease and lymphoma, and eventually, more effective treatments to alleviate symptoms, halt progression, and restore organ function. These procedures are linked to alterations that modify DNA, known as epigenetic modifications. These changes can affect gene expression and cell behavior. Researchers have tested several treatments for these processes, but many more are still in the discovery phase. In particular, the calculation of epigenetic risk scores offers the probability for improved classification of the subtypes of the disease and must be considered in subsequent clinical studies (155).

DNA methylation changes are being recognized as a crucial component of SjS genesis and progression (48). A notable observation is the limited connection between epigenetic signals and identified genetic risk loci in the context of SjS. This lack of concurrence implies that numerous genes contributing to the heightened risk of SjS likely operate upstream of cellular processes. These genes may trigger epigenetic alterations and disparities in gene expression without undergoing direct epigenetic modifications, presenting intriguing molecular targets for investigation.

Strong evidence implicates interferon pathways in SjS, as evidenced by disease associations with genetic variation in genes within these pathways. SjS patients show significant hypomethylation of ISGs. The majority of hypomethylation sites are related to augmented levels of ISGs. These observations confirm previous findings linking IFN activity to disease activity and emphasize the DNA methylation pattern stability (100, 103). Additionally, there is noteworthy hypomethylation observed in genes regulated by interferons. Genetic and epigenetic insights reinforce the robust correlation between the disease and the human leukocyte antigen (HLA) locus, a recognition spanning decades.

Epigenetic modifications exhibit variations across different tissues and cell types, yet a discernible pattern is beginning to surface within the context of SjS. This pattern entails extensive DNA methylation alterations, affecting T and B cells and the target tissue. A forthcoming challenge involves identifying drugs capable of selectively reverting these epigenetic changes. This sets them apart from currently available medications, which primarily operate in a nonspecific manner. Within immune cells and target tissues, other potential avenues for epigenetic treatment exist. These include modifying non-coding RNAs, altering histone acetylation patterns, and adjusting nucleosome positioning. In addition, the developing field of epitranscriptomics, involving post-transcriptional RNA modifications, shows potential. Although deserving attention, epitranscriptomics has not yet been explored within the realm of SjS. This aspect presents an exciting avenue for future research.

While numerous drugs under development are geared towards interferon pathways, treatments with a specific focus on antigen presentation or the induction of tolerance have not yet been effectively devised for SjS. Hydralazine and procainamide have been recognized to cause SS in rodents and humans by preventing DNA methylation since the 1990s. In light of this, Cole et al.’s observation of a tendency towards DNA hypomethylation in labial salivary gland (LSG) tissue from SjS patients is not surprising (113). The growing body of research confirming the association between DNA methylation and the clinical presentation of SjS individuals highlights the potential of DNA methylation as a clinical marker. The integration of DNA methylation markers may facilitate patient stratification according to disease subtypes. DNA methylation may be a valuable biomarker for monitoring the disease’s activity and response to treatment in certain diseases. In addition to the correlation of DNA methylation profiles with various health conditions, DNA methylation-based markers are useful for clinical applications due to the reliability and stability of DNA methylation and the ease of assessing DNA methylation patterns (156). There are currently several high-throughput methods available for studying DNA methylation on a large scale. Single-cell methods are now available to better understand DNA methylation and transcriptional changes in autoimmune rheumatic conditions. This poses new difficulties and opportunities and provides an opportunity to identify novel clinical indicators and therapeutic targets. The system based on CRISPR-Cas9 is also being investigated as a state-of-the-art tool for altering certain epigenetic variants, which has potential as a method for treating and preventing SjS.

Conclusively, the pathogenesis of SjS has been linked to targeting epigenetic dysregulation, for instance, changes in DNA methylation. These epigenetic modifications can profoundly impact gene expression profiles in immune cells, thereby contributing to the disease’s chronic inflammation and immune dysfunction. By intervening to rectify these aberrant DNA methylation patterns, it becomes possible to restore a more normalized gene expression landscape in immune cells, potentially mitigating the underlying causes of SjS. As immune cells like T and B cells are key players in the inflammatory and autoimmune response associated with SjS, modulation of DNA methylation in these cells may affect how they are activated, differentiated, and interact with other immune components. By restoring a more balanced immune response, targeting DNA methylation could offer the potential for lasting and meaningful improvements in patient well-being.

## Author contributions

YW: Conceptualization, Formal analysis, Methodology, Visualization, Writing – original draft. FR: Validation, Investigation, Methodology, Writing – original draft. WW: Formal analysis, Writing – original draft, Data curation, Funding acquisition, Software. XW: Supervision, Validation, Writing – review and editing. JT: Supervision, Validation, Writing – review and editing. JP: Writing – original draft. YL: Writing – original draft. ZW: Writing – original draft. SP: Writing – original draft. JS: Writing – original draft. YZ: Writing – original draft. HW: Writing – original draft. LY: Writing – original draft. FH: Writing – original draft.
